# A prospective study of repetition of self-harm following deliberate self-poisoning in rural Sri Lanka

**DOI:** 10.1371/journal.pone.0199486

**Published:** 2019-02-12

**Authors:** P. H. G. J. Pushpakumara, S. U. B. Thennakoon, T. N. Rajapakse, Ranil Abeysinghe, A. H. Dawson

**Affiliations:** 1 Department of Family Medicine, Faculty of Medicine and Allied Sciences, Rajarata University of Sri Lanka, Saliyapura, Sri Lanka; 2 Department of Community Medicine, Faculty of Medicine, University of Peradeniya, Peradeniya, Sri Lanka; 3 Department of Psychiatry, Faculty of Medicine, University of Peradeniya, Peradeniya, Sri Lanka; 4 Central Clinical School, University of Sydney, Sydney, Australia; University of Toronto, CANADA

## Abstract

**Introduction:**

Repetition of deliberate self-harm is an important predictor of subsequent suicide. Repetition rates in Asian countries appear to be significantly lower than in western high-income countries. Methodological differences in studies, and the impact of access to means of self-harm with comparatively higher lethality have been suggested as reasons for these reported differences. This prospective study determines the rates and demographic patterns of deliberate self-poisoning (DSP), suicide and repeated deliberate self-harm resulting non-fatal and fatal outcomes in rural Sri Lanka.

**Methods:**

Details of DSP admission in all hospitals (n = 46) and suicides reported to all police stations (n = 28) in a rural district were collected for the years 2011, 2012 and 2013. Demographic details of the cohort of patients admitted to all hospitals in 2011 due to deliberate self-poisoning (N = 4022), were screened to link with patient records and police reports of the successive two years with high sensitivity using a computer program. Then high specificity manual matching of all screened links was performed to identify repetition within 2 years of initial presentation. Life time repetition was assessed in a randomly selected subset of DSP patients (n = 433).

**Results:**

There were 15,639 DSP admissions, aged more than 9 years, and 1078 suicides during the study period. The incidence of deliberate self-poisoning and suicide in the population within the study area were 248.3/100,000 and 20.7/100,000 respectively, in 2012. Repetition rates at four weeks, one-year and two-years were 1.9% (95% CI 1.5–2.3%), 5.7% (95% CI 5.0–6.4) and 7.9% (95% CI 7.1–8.8) respectively. The median interval between two attempts were 92 (IQR 10–238) and 191 (IQR 29–419.5) days for the one and two-year repetition groups. The majority of patients used the same poison in the repeat attempt. The age and duration of hospital stay of individuals with repetitive events were not significantly different from those who had no repetitive events. The rate of suicide at two years following DSP was 0.7% (95% CI 0.4–0.9%). The reported life time history of deliberate self-harm attempts was 9.5% (95% CI 6.7–12.2%).

**Conclusions:**

The comparatively low rates of repetition in rural Sri Lanka was not explained by higher rates of suicide, access to more lethal means or differences in study methodology.

## Introduction

Deliberate self-harm (DSH) is a major global public health problem. The World Health Organization (WHO) projects that worldwide, the annual suicide mortality rate will increase to 1.53 million, and will constitute 2.4% of the total disease burden by 2020. [1]

A recent meta-analysis estimated that one in 25 patients presenting to hospital for self-harm will die by suicide in the next 5 years. [2] Understanding the factors that influence the rate and pattern of repetition of self-harm has the potential to inform prevention strategies and optimize follow-up after a self-harm episode. Geographic differences have been noted in 1-year non-fatal repetition rates. [2] European studies estimate the 1 year non-fatal repetition rate to be 17.1% (95% CI 15.9–18.4), whereas it is lower in Asia (10.0%, 95% CI 7.3–13.6). [2] Possible suggested reasons for the lower reported rates in Asian studies include methodological weakness (such as less reliable outcome data), higher lethality of self-poisoning and longer duration of hospital stay. [2] It has been suggested that identifying the reasons for this variation could provide insights into optimal configuration of health care services. [2]

Ingestion of poison or taking an overdose of drugs with suicidal intent accounts for more than 80% of deliberate self-harm in Sri Lanka. [3] This prospective study determines the rates of repeated self-harm at four weeks, one year and two year follow-up, which result in non-fatal or fatal outcomes, and estimates the life-time repetition rate of deliberate self-poisoning in a rural district of Sri Lanka.

## Methods

### Study setting and design

This study was conducted in the predominately rural agricultural district of Kurunegala (KD) in Sri Lanka. The district has a population of 1.6 million. [4] The population all have free access to 46 government hospitals, which include 45 district hospitals and 1 tertiary teaching hospital (Teaching Hospital Kurunegala/THK). [5] All in-hospital and community suicide deaths from any cause are reported to district police stations (n = 28).

A prospective cohort of all hospital presentations following deliberate self-poisoning (DSP) to government hospitals within the KD was established from 1^st^ January 2011 to 31^st^ December 2013, as part of a randomised control trial of the use of treatment guidelines for poisoning (Sri Lanka Clinical Trial Registry No. SLCTR/2010/ 008). Data was also collected on all suicides reported to district police stations. A randomly selected subset of patients and their bystanders were interviewed to determine rates of self-reported lifetime repetition.

### Recruitment of the prospective cohort to examine for repetition

Identification, demographic and clinical details of all DSP admissions were collected as part of the study. In THK all patients were enrolled into the cohort at the time of admission, by doctors employed as fulltime clinical research assistants. Patients were seen at least daily until discharge or death. In the other 45 district hospitals, data was extracted from patient medical records by postgraduate research assistants and entered into a study database, along with a scanned copy of the medical record of the patient’s admission. Hospitals were visited every 2–4 weeks depending upon the size of the hospital. Typically, all relevant admission records had been left aside at each hospital to facilitate case finding, but in each hospital the admission ledgers were also reviewed to ensure all relevant medical records had been identified. Any missing records were retrieved and entered into the database.

Details of all suicides reported to police stations during the study period were collected by visiting all 28 police stations in the district monthly. Data was retrieved from suicide registers at each police station, by postgraduate research assistants.

**Fig 1:**
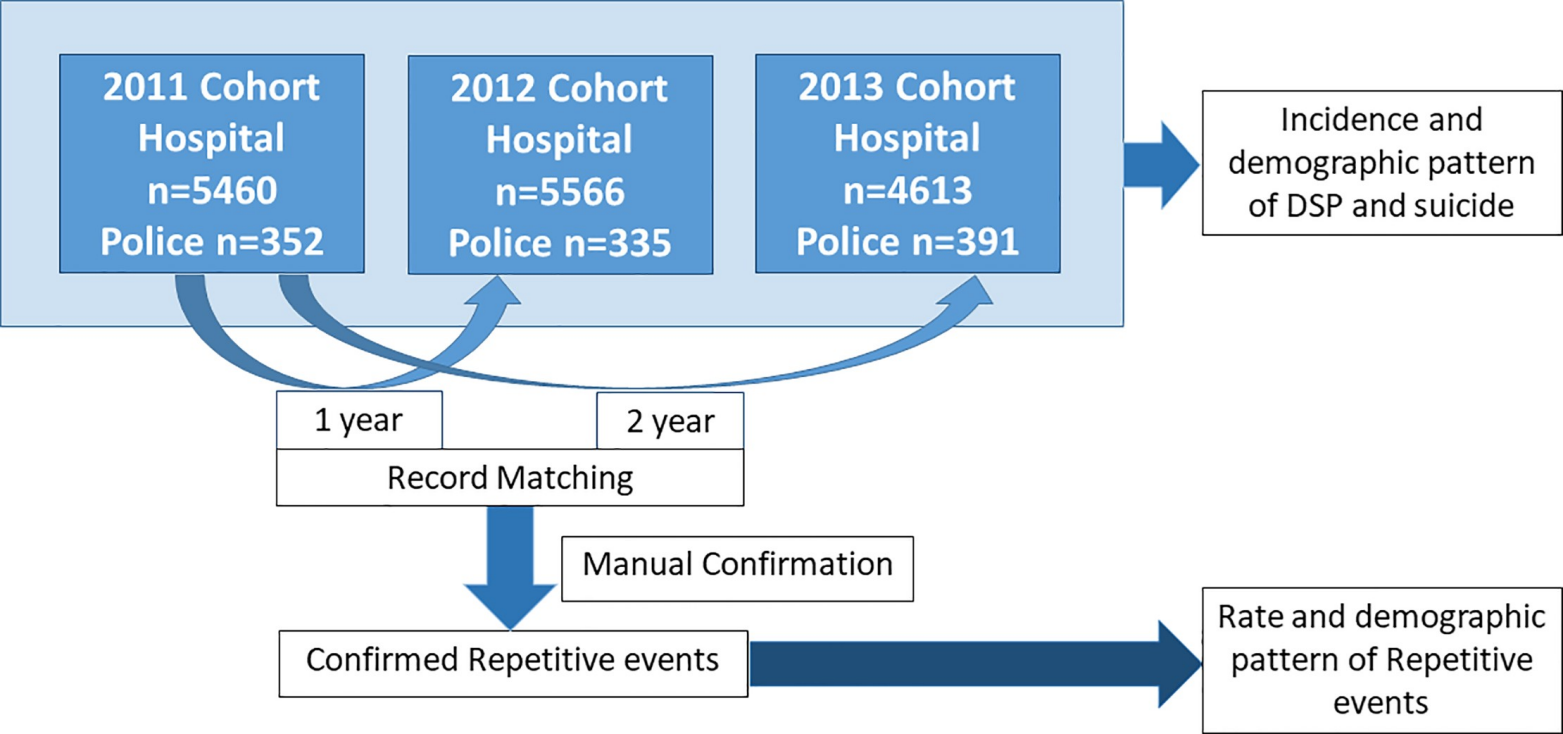
Recruitment and follow-up of the prospective repetition cohort. DSP admissions reported to all the hospital in KD and suicides reported to police stations were collected from 2011–2013. Data was collected regarding the incidence and demographic patterns of DSP and suicide. Details of the cohort of DSP patients presenting in 2011 (n = 4022) were matched with subsequent records of presentations in in 2012 and 2013 using a software with high sensitivity to identify repeat presentations. Manual confirmation was done in order to confirm repetitive events with a higher degree of specificity.

Within the cohort, the patients’ index admission was their first admission to any study hospital from 1^st^ January 2011 to 31^st^ December 2011. Following the index admission, the study database was examined for repeat presentations to hospitals or police stations, over a period of two years. As there is no unique patient medical record number within the provincial health system, identification of inter-hospital transfers and repeat presentations required individual identity linkage. After the linkage was established patients were de-identified and assigned a unique study number (Fig 1).

The initial linkage utilized the surname, at least one other name, sex and age as mandatory fields and residential address as an optional field, for confirmation of matching. A five step method, which has been adapted from a English-Sinhala transliteration system [6] and a process of matching names in Sinhala, [7] was used in screening to generate possible spelling combinations of surname, other names and village/address. It consisted of transliteration from English into Sinhala, decomposition of Sinhala words, single and multi-character replacements, generation of possible spelling combinations of Sinhala words by combining replaced characters and transliteration into English. Records were linked using a semi-automated stepwise data matching and filtering process. A high sensitivity algorithm was used to generate a list of potential linked records from the study database, and these potential links were then each examined manually for final confirmation of the link.

### Recruitment of cohort to examine for lifetime recalled previous self harm

Data on lifetime recalled previous self-harm was conducted in a randomly selected cohort of patients admitted to THK following DSP. Patients were randomly selected using a computer program, from blocks of 7 consecutively admitted consenting DSP patients during the consecutive eighteen months from 1^st^ July 2011 onwards. Immediately prior to discharge these patients were invited to participate in a structured interview conducted by trained medical graduate research assistants. Patients were asked about previous episodes of self-harm, by any method. Information obtained from the interview was verified via a close relative or someone who knew the patient well.

### Data analysis

Data were entered into a Microsoft Access database and analysed using IBM SPSS Statistics for Windows, Version 23.0. Medians, quartiles and percentages with confidence interval at 95% significant level were calculated to describe variables. Odds ratios and confidence interval at 95% significant level were calculated for categorical data. The one-sample binominal test was applied to examine the distribution of a single dichotomous variable. The Mann Whitney U test was used to compare differences between two independent groups when the dependent variable was either ordinal or continuous and not normally distributed. The one-sample Kolmogorov-smirnov test was applied to compare a sample with a reference probability distribution. The acceptance of level of significant was 0.05.

### Ethics statement

Ethics approval was obtained from Ethical Review Committee of the Faculty of Medicine, University of Peradeniya for “A clustered RCT of educational interventions on treatment of patients with acute poisoning in rural Asian hospitals”. Ethical approval for additional data collection was obtained from the human ethics review committee of the Faculty of Medicine and Allied Sciences, Rajarata University of Sri Lanka. This included approval for the initial record linkage using personal identification details and also approval for obtaining information from a third party. The study was conducted with the support of the Provincial Department of Health Care and nutrition, of the North Western Province, and Department of Police, Sri Lanka.

## Results

### Prospective cohort study

A total of 15,639 DSP admissions aged more than 9 years, and 1,078 records of suicide were collected from hospitals and police stations respectively, during the 3 years. After removal of inter-hospital transfers, to avoid to double counting, there were 11,125 unique DSP events. THK received 53.3% (n = 5,928) of all DSP cases in the district either as a direct admission or following transfer. The DSP incidence and male female ratio for 2012 were calculated using the census data for 2012 (Table 1). Within the cohort the three most frequent toxins ingested were: agro-chemicals (35.5% 95% CI 34.7–36.2), medications (32.9% 95% CI 32.1–33.6), and oleander seeds (15.2% 95% CI 14.6–15.7).

**Table 1:** The incidence of DSP in 2012 in the Kurunegala District among males and females, with male to female ratio.

Age (Years)	DSP Incidence in 2012 per 100,000 Population* (95% CI)			Male: Female Ratio
	Male	Female	Total	
10–14	80.7 (58.3–103.1)	188.9 (154.3–223.4)	134.3 (113.8–154.8)	0.4
15–19	634.4 (570.9–697.8)	1304.0 (1213.6–1394.3)	971.4 (916.1–1026.7)	0.5
20–24	769.6 (694.3–844.9)	760.2 (688.0–832.4)	764.7 (712.6–816.9)	1.0
25–29	414.4 (360.7–468.1)	371.3 (323.6–418.9)	391.5 (355.8–427.2)	1.1
30–34	317.2 (272.6–361.7)	231.5 (195.2–267.9)	272.4 (243.9–300.9)	1.4
35–39	292.2 (247.3–337.0)	144.0 (113.4–174.6)	216.0 (189.1–242.9)	2.0
40–44	243.3 (201.8–284.8	88.8 (64.4–113.1)	163.8 (140.1–187.6)	2.7
45–49	231.2 (189.9–272.6)	56.7 (37.1–76.4)	140.4 (118.0–162.7)	4.1
50–54	175.4 (138.7–212.0)	54.2 (34.8–73.6)	111.8 (91.6–132.0)	3.2
55–59	146.2 (110.6–181.7)	23.5 (10.2–36.8)	80.6 (62.6–98.6)	6.2
60–64	118.0 (83.1–152.9)	36.2 (18.4–53.9)	73.6 (55.0–92.2)	3.3
65–69	125.6 (78.2–172.9)	36.3 (13.8–58.8)	75.5 (51.1–99.8)	3.5
70–74	128.6 (70.8–186.4)	15.3 (-2.0–32.7)	64.0 (37.3–90.8)	8.4
75–79	120.4 (49.3–191.6)	14.2 (-5.5–34.0)	56.1 (25.6–86.6)	8.5
80 & over	188.8 (99.0–278.6)	37.1 (4.6–69.6)	97.8 (56.9–138.7)	5.1
Overall	257.7 (246.4–269.0)	239.5 (229.1–249.9)	248.3 (240.6–255.9)	1.1

*The incidences of DSP were calculated based on the 2012 DSP events

The follow-up cohort consisted of 4,022 (50.8% males and 49.2% females) unique patients who presented with DSP in 2011, with a median age of 23 years. A total of 77 (n = 44, 57% were males) had a repeat self-harm event within the first four weeks from the indexed event. The overall repetition rates were: 1.9% (95% CI 1.5–2.3%) at four-weeks, 5.7% (95% CI 5.0–6.4) at 1 year and 7.9% (95% CI 7.1–8.8) at 2 years (Table 2). Of the patients who repeated DSP: 290 (91.2%) had only one repetitive attempt, 24 (7.5%) had two, 3 (0.9%) had three and one (0.3%) had four during this period.

**Table 2:** Rates of repetition at four weeks, one year and two years, by age and sex.

Age (Years)	Repetition rate as a % (95% CI) [n]								
	4 Weeks			1 Year			2 Years		
	Male	Female	All	Male	Female	All	Male	Female	All
10–14	2.4 (-2.2–7.0) [1]	0 (0–0) [0]	0.8 (-0.7–2.2) [1]	2.4 (-2.2–7.0) [1]	3.3 (-0.4–7.0) [3]	3.0 (0.1–5.9) [4]	2.4 (-2.2–7.0) [1]	6.7 (1.5–11.8) [6]	5.3 (1.5–9.1) [7]
15–19	1.0 (0.0–2.0) [4]	2.2 (1.1–3.3) [17]	1.8 (1.0–2.5) [21]	6.8 (4.3–9.3) [27]	6.3 (4.6–8.1) [47]	6.5 (5.0–7.9) [74]	10.1 (7.1–13.0) [40]	9.3 (7.2–11.5) [69]	9.6 (7.9–11.3) [109]
20–24	2.1 (0.7–3.5) [8]	0.5 (-0.2–1.2) [2]	1.3 (0.5–2.0) [10]	5.2 (3.0–7.5) [20]	5.1 (3.0–7.3) [21]	5.2 (3.6–6.7) [41]	7.9 (5.2–10.6) [30]	6.9 (4.4–9.3) [28]	7.3 (5.5–9.2) [58]
25–29	1.1 (-0.1–2.3) [3]	2.4 (0.5–4.3) [6]	1.7 (0.6–2.9) [9]	6.3 (3.4–9.2) [17]	4.4 (1.9–7.0) [11]	5.4 (3.4–7.3) [28]	8.1 (4.9–11.4) [22]	6.1 (3.1–9.0) [15]	7.1 (4.9–9.4) [37]
30–34	3.0 (0.6–5.4) [6]	2.4 (0.1–4.7) [4]	2.7 (1.1–4.4) [10]	6.6 (3.1–10.0) [13]	3.6 (0.8–6.4) [6]	5.2 (2.9–7.5) [19]	9.1 (5.1–13.1) [18]	4.8 (1.5–8.0) [8]	7.1 (4.5–9.7) [26]
35–39	2.2 (0.1–4.3) [4]	2.1 (-0.8–5.1) [2]	2.2 (0.5–3.9) [6]	8.8 (4.7–13.0) [16]	5.4 (0.8–10.0) [5]	7.7 (4.5–10.8) [21]	11.0 (6.5–15.6) [20]	5.4 (0.8–10.0) [5]	9.1 (5.7–12.5) [25]
40–44	0.8 (-0.8–2.4) [1]	0 (0–0) [0]	0.6 (-0.5–1.7) [1]	2.5 (-0.3–5.2) [3]	2.0 (-1.8–5.8) [1]	2.3(0.1–4.6) [4]	5.8 (1.6–9.9) [7]	3.9 (-1.4–9.2) [2]	5.2 (1.9–8.6) [9]
45–49	2.7 (-0.3–5.7) [3]	4.9 (-1.7–11.5) [2]	3.3 (0.4–6.1) [5]	7.2 (2.4–12.0) [8]	4.9 (-1.7–11.5) [2]	6.6 (2.6–10.5) [10]	7.2 (2.4–12.0) [8]	9.8 (0.7–18.8) [4]	7.9 (3.6–12.2) [12]
50–54	4.7 (0.7–8.7) [5]	0 (0–0) [0]	3.5 (0.5–6.5) [5]	8.5 (3.2–13.8) [9]	2.8 (-2.6–8.1) [1]	7.0 (2.8–11.2) [10]	12.3 (6.0–18.5) [13]	2.8 (-2.6–8.1) [1]	9.9 (4.9–14.8) [14]
55–59	5.1 (-0.5–10.7) [3]	0 (0–0) [0]	4.0 (-0.4–8.5) [3]	11.9 (3.6–20.1) [7]	6.7 (-5.9–19.3) [1]	10.8 (3.7–17.9) [8]	18.6 (8.7–28.6) [11]	6.7 (-5.9–19.3) [1]	16.2 (7.8–24.6) [12]
60–64	9.4 (-0.7–19.5) [3]	0 (0–0) [0]	7.3 (-0.6–15.3) [3]	12.5 (1.0–24.0) [4]	0 (0–0) [0]	9.8 (0.7–18.8) [4]	12.5 (1.0–24.0) [4]	0 (0–0) [0]	9.8 (0.7–18.8) [4]
65–69	0 (0–0) [0]	0 (0–0) [0]	0 (0–0) [0]	4.5 (-4.2–13.2) [1]	0 (0–0) [0]	2.9 (-2.7–8.6) [1]	4.5 (-4.2–13.2) [1]	0 (0–0) [0]	2.9 (-2.7–8.6) [1]
70–74	0 (0–0) [0]	0 (0–0) [0]	0 (0–0) [0]	0 (0–0) [0]	0 (0–0) [0]	0 (0–0) [0]	5 (-4.5–14.5) [1]	0 (0–0) [0]	4.2 (-3.8–12.2) [1]
75–79	20 (-15.1–55.1) [1]	0 (0–0) [0]	11.1 (-9.4–31.6) [1]	20 (-15.1–55.1) [1]	0 (0–0) [0]	11.1 (-9.4–31.6) [1]	20 (-15.1–55.1) [1]	0 (0–0) [0]	11.1 (-9.4–31.6) [1]
80 & over	50 (1–99) [2]	0 (0–0) [0]	22.2 (-4.9–49.4) [2]	50 (1–99) [2]	0 (0–0) [0]	22.2 (-4.9–49.4) [2]	50 (1–99) [2]	0 (0–0) [0]	22.2 (-4.9–49.4) [2]
Total	2.2 (1.5–2.8) [44]	1.7 (1.1–2.2) [33]	1.9 (1.5–2.3) [77]	6.4 (5.3–7.4) [129]	5.0 (4.0–5.9) [98]	5.7 (5.0–6.4) [227]	8.8 (7.6–10.1) [179]	7.0 (5.9–8.2) [139]	7.9 (7.1–8.8) [318]

**Fig 2:**
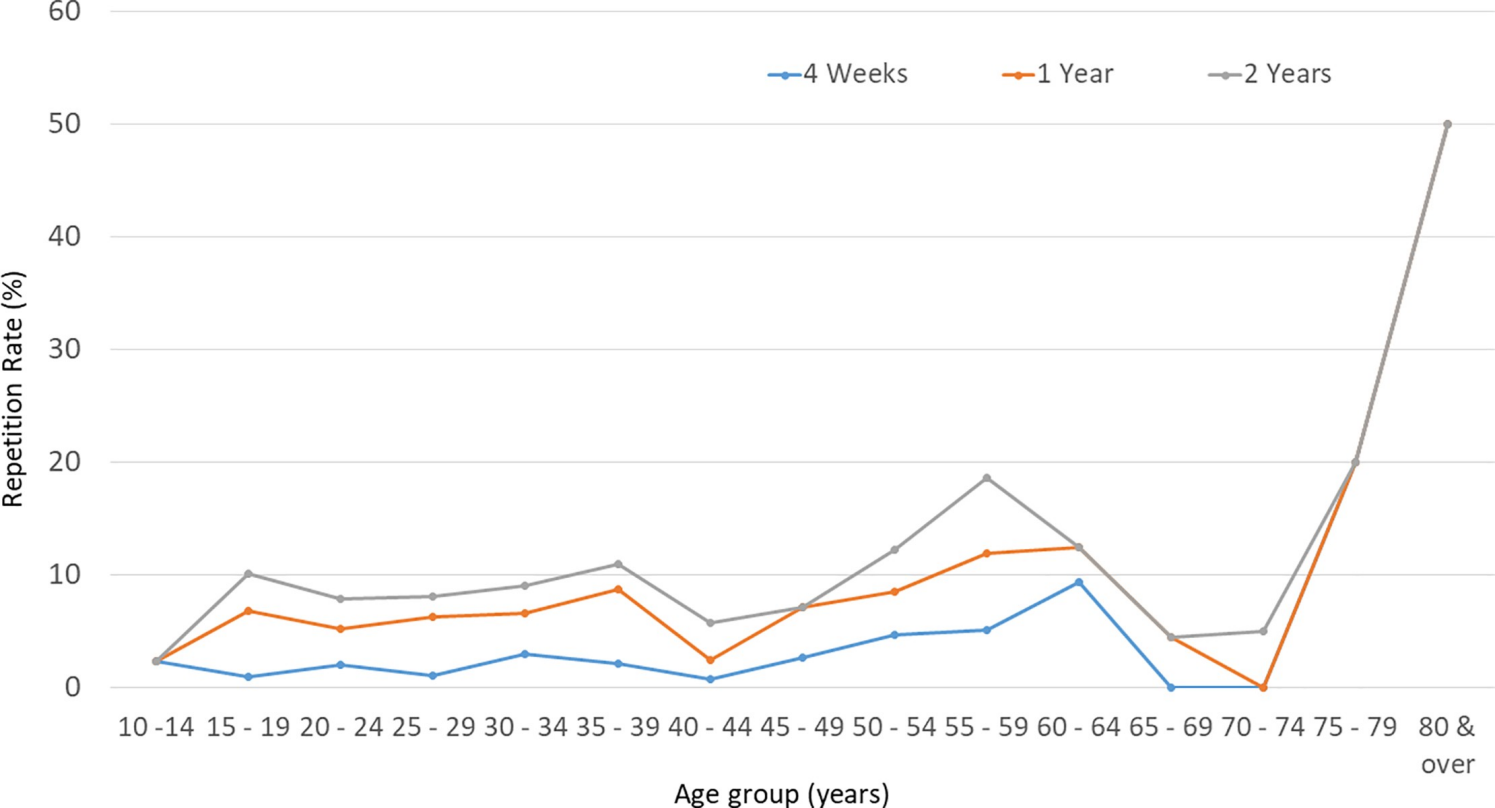
Rate of repetition among males, by age group, at four weeks, one year and two years. Repetition rate, 4 weeks: Number of males in a particular age group with repetitive events within 4 weeks from the indexed event/Male DSP patients in the particular age group of 2011 cohort. One-year: Number of males in a particular age group with repetitive events within a year from the indexed event/Male DSP patients in the particular age group of 2011 cohort. Two-year: Number of males in a particular age group with repetitive events within two years from the indexed event/Male DSP patients in the particular age group of 2011 cohort.

**Fig 3:**
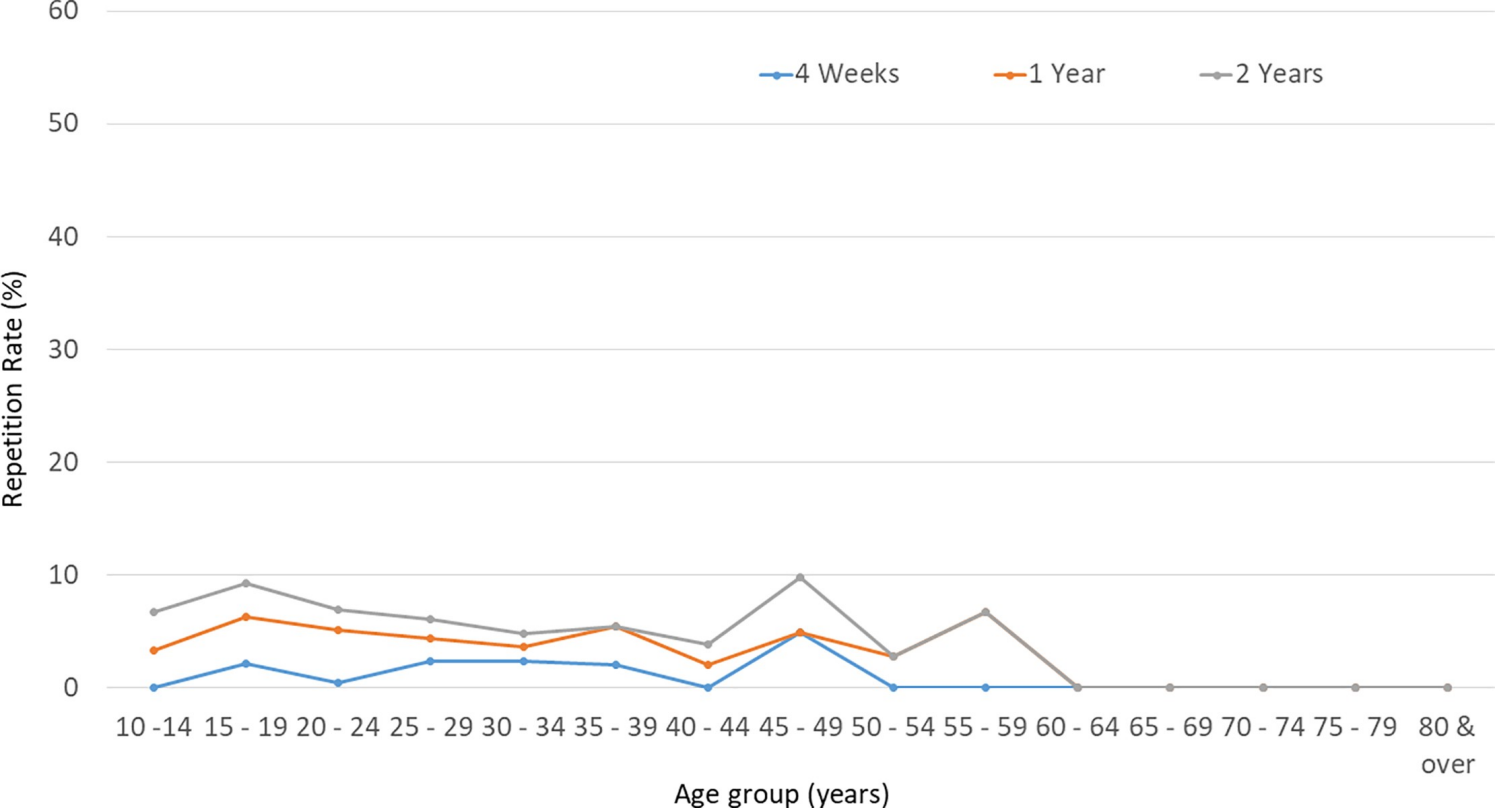
Rate of repetition among females, by age group, at four weeks, one year and two years. Repetition rate, 4 weeks: Number of females in a particular age group with repetitive events within 4 weeks from the indexed event/Female DSP patients in the particular age group of 2011 cohort, One-year: Number of females in a particular age group with repetitive events within a year from the indexed event/Female DSP patients in the particular age group of 2011 cohort, Two-year: Number of females in a particular age group with repetitive events within two years from the indexed event/Female DSP patients in the particular age group of 2011 cohort.

The repetitive events were more common among males (p = 0.03: one-sample binominal test), with an odds ratio for repetitive attempts of 1.3 (95% CI 1–1.6). The median age of males who repeated self-harm within the two-year follow-up period was 28 years (IQR 20–40 years) and for females it was 19 years (IQR 16–25 years). Repetition rates were highest in the 15–19 year age group, for both sexes. (Table 2, Figs 2 and 3) Within the repetition group, females were younger than males (p < 0.0001 Mann Whitney U test). There was no significant difference in the median age of non-repetition (23 years, IQR 18–33 years) and repetition individuals (22 years, IQR 18–35 years, p = 0.60 Mann Whitney U test).

**Fig 4:**
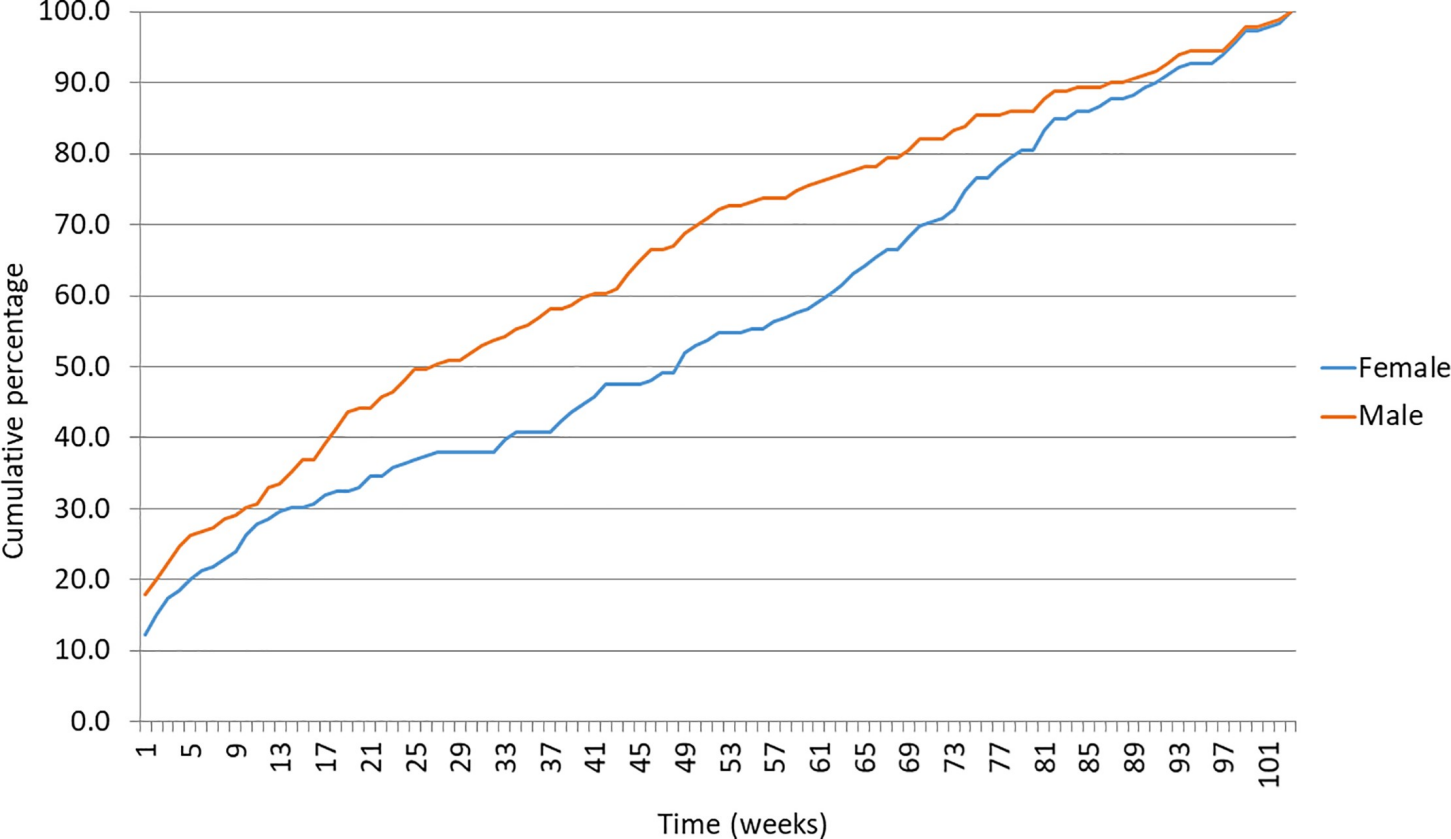
The cumulative probability of the first repetitive DSP event during the first two years, among males and females. Percentages of repetitive events occurred in each week after the index event.

The average intervals between two consecutive events were 246.8 days (SD 223.4) among males and 238.5 days (SD 207.0) among females, and this difference was not significant, p = 0.7. The intervals between index event and the first repetitive event were not normally distributed, p < 0.0001 (one-sample Kolmogorov-smirnov test). The highest risk for repetition was observed in the initial one week period, where 17% (n = 54) of repetitive attempts occurred (Fig 4). 9.1% (n = 29) had re-attempted on the following day of the indexed event. Median times for repetition within 1 year and 2 years were, 92 (IQR 10–238) and 191 (IQR 29–419.5) days respectively.

Agro-chemical and oleander use in repetitive events were higher than their use in the indexed cohort. The first repetitive self harm event had a fatal outcome in 28 (8.8%) individuals, of whom all were males. The outcome with regards to all second, third or fourth repetitive self-harm events were non-fatal. The two year rate for suicide following DSP was 0.7% (95% CI 0.4–0.9%). The mean age for those who carried out fatal repetitive events was 49.7 (SD 15.3), and this group was significantly older than those with a non-fatal outcome, p<0.0001. Nearly 40% of the fatal two year repetitive events occurred within the first week, and 50% within the first 3 weeks following the indexed event. Fatal repetitions were most commonly due to poisoning (24/28), (Table 3) but there was one fatality due to hanging and 3 were not classified.

**Table 3:** Fatal and non-fatal repetitive events reported to hospitals by the type of the poison and pattern of use at subsequent events.

Type of poison ingested	Individuals in Cohort	Individuals with Repetitive events			
		Use at the indexed Event	Same Method used in any two consecutive events	Used for the fatal events	Same Method used in the fatal and Indexed event
	% (95% CI)	n (%)	n (%)	n (%)	n (%)
Agro-Chemicals	36.2 (35.4–36.9)	127 (39.9)	76 (48.7)	22 (91.7)	17 (94.4)
Medicine	32.9 (32.2–33.6)	93 (29.2)	44 (28.2)	0 (0)	0(0)
Oleander	15.2 (14.6–15.8)	60 (18.9)	33 (21.1)	2 (8.3)	1 (5.6)
Hydrocarbon	4.6 (4.3–4.9)	10 (3.1)	2 (1.3)	0 (0)	0 (0)
Other	3.6 (3.3–3.9)	9 (2.8)	0 (0)	0 (0)	0 (0)
Unknown	8.2 (7.8–8.6)	19 (6)	0 (0)	0 (0)	0 (0)
Total	100	318 (100)	155 (100)	24 (100)	18(100)

The median hospital stay of DSP patients was two days for all types of poisons, and there was no significant difference between repetition and non-repetition patients (Table 4).

**Table 4:** Duration of hospital stay and case-fatality ratio by type of poison.

Type of poison	Cases	Deaths	Case fatality ratio	Median Hospital stay (IQR) in days			
				Peripheral		THK	
				Non-repetitive	Repetitive	Non-repetitive	Repetitive
Agro-Chemical	5092	136	2.67%	2 (2–4)	3 (2–4.5)	2 (2–3)	2 (2–3)
Medicine	5014	8	0.16%	2 (1–3)	2 (1.75–3)	2 (1–2)	2 (1–2)
Oleander	2814	32	1.13%	2 (2–3)	3 (2–4)	2 (2–3)	3 (2–4)
Hydrocarbon	623	3	0.48%	2 (1–2)	2 (1.25–2.75)	2 (1–2)	2 (1–2)
Acid /Alkali	35	1	2.86%	1 (1–2)	-	2 (2–3)	-
Rodenticide	92	0	0%	2 (1–3)	-	2 (2–2)	2 (2–2)
Fertilizers	66	0	0%	2 (1–3)	3 (3–3)	2 (2–2)	-
Other/Combinations	139	0	0%	2 (1–2)	2 (1–2)	2 (2–3)	2 (2–2)
Unknown	2039	18	0.88%	2 (1–2)	1 (1–2)	2 (1–3)	3.5 (2.25–6.25)
Total	15914	198	1.24%	2 (1–3)	2 (2–3)	2 (2–3)	2 (2–3)

There were 1,078 suicides in the district by all methods in 2011 to 2013 (Table 5). The incidence of suicide in KD was 20.7/100,000 (95% CI 18.5 to 22.9) and the male: female ratio was 4.4 (Table 6). Poisoning accounted for 646 of suicides, and within the poisoning group only 31.2% of male and 33.3% of female deaths occurred in hospitals.

**Table 5:** Suicides reported to police stations in KD by method and sex.

Methods	Male		Female		Total	
	n	%	n	%	n	%
Burning	5	0.6	7	3.9	12	1.1
Stabbing/Cutting with a sharp weapon	2	0.2	1	0.6	3	0.3
Drowning	24	2.7	18	10.1	42	3.9
Gun shot	2	0.2	0	0.0	2	0.2
Hanging	269	29.9	30	16.9	299	27.7
Jump to motor vehicle	1	0.1	0	0.0	1	0.1
Jump to Train	43	4.8	7	3.9	50	4.6
Oleander	35	3.9	27	15.2	62	5.8
Agro-chemicals	499	55.4	84	47.2	583	54.1
Agro-chem. & Drowning	1	0.1	0	0.0	1	0.1
Other	11	1.2	4	2.2	15	1.4
Not Recorded	8	0.9	0	0.0	8	0.7
Total	900	100.0	178	100.0	1078	100.0

**Table 6:** Age standardized suicide incidences in KD in 2012 among males and females.

	Age adjusted Suicide Incidence per 100,000 population* (95% CI)			Male: Female Ratio
	Male	Female	Total	
10–14	0.0	4.9 (-0.6 to 10.5)	2.4 (-0.3 to 5.2)	-
15–19	13.2 (4.1 to 22.4)	14.7 (5.1 to 24.2)	13.9 (7.3 to 20.6)	0.9
20–24	40.3(23.1 to 57.5)	19.6 (8.0 to 31.2)	29.6 (19.3 to 39.8)	2.1
25–29	25.3 (12.1 to 38.6)	1.6 (-1.5 to 4.7)	12.7 (6.3 to 19.1)	15.9
30–34	26.0 (13.3 to 38.8)	17.8 (7.7 to 27.9)	21.7 (13.7 to 29.8)	1.5
35–39	25.1 (11.9 to 38.2)	6.8 (0.1 to 13.4)	15.7 (8.4 to 22.9)	3.7
40–44	47.9 (29.5 to 66.3)	5.2 (-0.7 to 11.1)	26.0 (16.5 to 35.4)	9.2
45–49	67.4 (45.1 to 89.8)	8.9 (1.1 to 16.6)	36.9 (25.5 to 48.4)	7.6
50–54	77.7 (53.3 to 102.1)	3.6 (-1.4 to 8.6)	38.9 (27.0 to 50.7)	21.5
55–59	45.0 (25.3 to 64.7)	0.0	20.9 (11.8 to 30.1)	-
60–64	75.1 (47.3 to 102.9)	11.3 (1.4 to 21.2)	40.5 (26.7 to 54.3)	6.6
65–69	74.4 (37.9 to 110.9)	10.9 (-1.4 to 23.2)	38.8 (21.3 to 56.2)	6.8
70–74	108.3 (55.2 to 161.3)	25.5 (3.1 to 47.9)	61.1 (34.9 to 87.3)	4.2
75–79	76.6 (19.9 to 133.4)	7.1 (-6.8 to 21.1)	34.5 (10.6 to 58.4)	10.8
80 & over	77.7 (20.1 to 135.3)	0.0	31.1 (8.1 to 54.2)	-
Total	34.6 (30.5 to 38.7)	7.8 (5.9 to 9.7)	20.7 (18.5 to 22.9)	4.4

*Suicide incidences were calculated based on the suicides occurring in 2012

### Lifetime recalled repetition study

The lifetime rate of previous DSH was studied in 433 (male 47% and female 53%) randomly selected cases. Forty one (9.5%) had a life time history of DSH attempts; 20 (48.8%) males and 21 (51.2%) females. The average age of cases who had made previous attempts was 26.9 years (SD 13.1, 95% CI 22.8–31.1). Amongst the cases who had made previous attempts, a majority had made only one previous attempt (32, 78%), the remainder had made two previous attempts (8, 19.5%) and one patient had four previous attempts.

## Discussion

The findings of the present study indicate that both the self-reported recalled life-time and record based two-year repetition rates are less than 10%. The life time repetition rate is higher compared to one year or two year repetition rate because repetitive attempts can occur at any point of the life. [8–10] Another potential reason for this is that the life time repetition rate was based on a referred hospital sample, which may have introduced a referral bias for patients with higher intent, whereas other rates were calculated for the entire KD, including patients presenting to primary rural hospitals many of who were not transferred to referral hospitals.

Almost all the previous studies conducted in Sri Lanka have been based on self-reported, life-time, recalled, repetition rates. Though the method is different in this study, the self-reported life-time repetition rate of KD is close to the value reported from socio-economically similar agricultural areas published in a previous studies, of 8.7% in North-Central Province (NCP)[11] and 7% in the Central and North Western Provinces. [8,12] Two psychological autopsy studies conducted in the NCP [13] and Rathnapura [14] reported a higher lifetime value, of 26%. A telephone interview based study conducted at Teaching Hospital Peradeniya, Sri Lanka, reported recalled one year repetition rate, 2.7%. [15] Contextual and methodological differences partially explain these difference in rates.

The majority of repetitive attempts was in males, consistent with a previous study in another district. [11] However, there was no significant difference across genders according to a European study. [16] In our study, all deaths from repetition of self harm was reported among males. A systematic review on repetition has shown that there are no definitive characteristics of an index episode of self-harm that are strong predictors of repetition, compared to patient factors such as long-standing psychosocial vulnerabilities, which are more important. [17]

In our study, the risks of repetition was higher in the initial post event period. The median times to repetition within 1 year and 2 years were around three and six months respectively comparable to the median time of 105 days in a Taiwan study of 1 year repetition. [18] The risk for repetition is highest in the first 3 to 6 months after a suicide attempt but remained substantially elevated from the general population for at least 2 years. [8–10]

A recent meta-analysis has reported that the estimated one year non-fatal repeat self-harm rate is considerably lower in Asian countries compared to Western countries, 10% vs 16.3% [2]. Western countries report higher repetition rates despite having better developed medical, psychological and social services than most Asian countries. It is possible that this is due to better ascertainment of cases gained through utilizing better medical records. However, with the robust methodology, our study’s results reconfirms lower rates of repetition in Asia with considerable accuracy. Rates of repetition resulting in deaths are comparable in our study with those seen in the west.

Risk factors for suicidal behaviour influence the risk of repetition, and these factors may be different among Asians [19]. Lower repetition frequencies have been reported among non-Western immigrants in a study conducted in seven European countries [20]. This suggests that cultural factors may have important influences on repetition. Potential factors that require further research could include stronger extended family structures which may offer more support, and stronger social structures within small communities. Such support may be particularly important in areas similar to our study area where there is a lack of outreach community services and mental health services. This lack of services may facilitate increased engagement of the family with the patient for at least for a short period and thus may prevent future events. [21,22] Continuing extended family support may also be a factor that helps to keep lifetime repetition rate at a lower level, and this has been described as a potent psycho-therapeutic factor in the Indian context. [23,24]

Examination of culture, gender and suicidal behaviour in Sri Lanka has suggested that both emotion focused and problem focused support is deemed needed for people who have attempted suicide, with a greater emphasis on emotion-focused support for females. [25] Continuing family support throughout the adolescent years and after marriage through the extended family is an integral part of the Sri Lankan culture. The majority of school adolescents perceive their families as intimate and close (60%) and considered family as refuge (52%) for a problem. [26] In Sri Lanka individuals give a higher priority to the family’s requirements compared to their own needs. This may ensure the emotional warmth and bonds among family members. Social support is a well known protective factor for suicidal behaviour and may contribute to the observed lower repetition rates. [27–29]

The hospital experience following a DSH attempt period may have an effect on reducing repetitive attempts. In this study the median hospital stay of 2 days is longer than the one day stay reported in western countries. [2,30,31] In England, half of the patients with self-harm presenting to the emergency department were discharged without being admitted to hospital. [32] In contrast, same day discharges were limited to 3% and 4% in THK and peripheral hospitals. A short hospital stay may allow the patient to be discharged back into to the same environment that contributed to the suicidal behaviour. This study and others have shown that the initial post attempt period carries the highest risk for repetition. [33] A longer length of hospital stay may contribute to lower repetition rates by providing a safer environment in this high risk period allowing the patient and family time to calm down and organize a post-discharge plan. [11]

A suggested explanation for the observed lower repetition rate was a higher case fatality of the first episode. [11] This explanation is based on an assumption that individuals at risk of repetition will have high suicidal intent and choose methods with higher potential lethality. However, in Sri Lanka, self-harm is often impulsive and the ingested poison is chosen on the basis of availability with little knowledge of potential lethality. [34] In addition there has been a significant reduction in case fatality rates over time, whereas repetition rates have remained low. [35] A worst case analysis is to assume all 221 poisoning suicides reported to police stations in 2011 were also due to a repetition attempt within a year of an index event. This worst case scenario would produce 448 repetition cases within a year with a one year repetition rate of 11.1% (95% CI: 10.2% -12.1%) a value still much lower to the one year repetition rate in Europe.

The population based DSP incidence reported in our study area is considerably lower than that observed by Knipe et. al. [35] This difference is most likely due to double counting of DSP due to high rates of inter-hospital transfer [36] that artificially in inflates the incidence. When transfers were included and double counted, the DSP incidence increases to 347.4/100,000 (95% CI 338.3–356.4) in KD. In contrast to the incidence of suicide, the incidence of DSP was slightly higher among males compared to females, and the male to female ratio was 1.1:1 which is exact opposite of the sex ratio of the district’s population. [37] This finding is compatible with the previous findings, that sixteen out of seventeen studies reported higher male to female gender ratio for DSH. [38] The pattern observed for age standardized DSP incidents is different to the pattern of suicide. One third of DSP occurred in 15–24 year age group and more than half were aged less than 34 years. This is in keeping with the national pattern for Sri Lanka, [39] as well as patterns in the South East Asian region. [40,41]

The incidence of suicide in KD has remained stable over the last decade; it was 20.7/100,000 in 2012, and the average value for the 2001–2006 period was 21.3/100,000 [39,42], in the presence of rising trends of DSP. [35] This observation can be explained by three main mechanisms; (1) continuum of the reduction observed from 1996 with measures taken in the 1990s, such as restriction of the import and sale of WHO Class I toxicity pesticides and decriminalization of suicide, (2) improvement of medical management of self-poisoning, and, (3) shifting of methods from lethal pesticides to less lethal medicaments. [35,43] The male to female ratio of suicide incidence is similar to Europe and countries in the American subcontinent, rather than Asian countries. [40] The age standardized pattern of suicide incidence is similar to national [39] and international statistics. [44–48] Poisoning with agro-chemicals is still the major means of suicide in rural agricultural Sri Lanka in contrast to suicide by hanging, which is the more common method overall, based on national level data. [39] In addition, more than three forth of individuals who ingested agro-chemicals at the fatal repetitive event used the same method at the index event. However, researchers should be careful in interpreting hospital based suicide data where more than two third of suicides due to poisoning are not reported to hospitals.

Though there is a considerable amount of literature available to explain the risk factors that are responsible for higher rates of DSH in Sri Lanka, culture specific protective factors that lead to lower repetition rates are poorly explored. These protective factors should be further explored to explain the lower rates of repetition. These protective factors may provide a base to promising preventive strategies of DSH. Further, measures directed for prevention of repetition alone may not produce considerable impact in preventing suicidal behaviour, in the presence of lower repetition rates.

### Limitations

Data collection was conducted only in government hospitals. Less severe cases, and those who may have presented to the private-sector or out-patient-care services might have been missed; however previous surveys have reported that admission due to self poisoning to rural private hospitals in Sri Lanka is extremely rare.

In calculating record based one and two year repetition rates, only the DSP admissions were considered for non-fatal events; not considering other methods of DSH, might have had an effect on lower rates. It has been suggested that individuals who attempt self-injury are more prone to repetitive attempts compared to those who attempt self-poisoning. [49] However, this effect may not be significant because more than 80% of DSH in Sri Lanka are due to poisoning and over dosage of medicines [12], and all fatal events were considered.

There are no unique patient identifiers in the provincial or national health system in Sri Lanka. The source of information, for patient details at the point of patient registration is the guardian and/or patient, and there is no verification or cross-checking. Due to associated stigma, patients might provide incomplete information to hide their identity. Therefore, the validity and reliability of the identification details in heath records may be limited. This may have affected the reliability of repetition matching process.

## Conclusions

The rate of repetition of DSH in Sri Lanka is very low compared to Western countries and other countries in the region. Therefore measures directed towards prevention of repetition alone may not produce a significant impact in preventing suicidal behaviour. Culture specific protective factors that lead to lower repetition rates should be further explored and they may provide a base for promising preventive strategies of DSH.
